# From Identification to Sensing: RFID Is One of the Key Technologies in the IoT Field

**DOI:** 10.3390/s22197523

**Published:** 2022-10-04

**Authors:** Yvan Duroc

**Affiliations:** UMR5005, Ampère, CNRS, Université Claude Bernard Lyon 1, INSA Lyon, Ecole Centrale de Lyon, Univ Lyon, 69130 Ecully, France; yvan.duroc@univ-lyon1.fr

**Keywords:** healthcare, identification, Internet of Things, RFID, RFID sensor tag

## Abstract

RFID (radio frequency identification) technology appeared nearly 70 years ago. Deployed more widely only from the early 2000s, it is now booming and its development is still accelerating. As its name indicates, its original function was the identification (of objects, animals, people) and its applications were then essentially aimed at traceability, access control and logistics. If this type of use is still relevant today with more and more new application contexts and more and more efficient RFID tags, RFID has also evolved by integrating new capabilities. These new tags, known as augmented tags, include an information capture function. With the explosion of connected objects and the emergence of the Internet of Things (IoT), this old technology that is RFID still has a promising future and will probably be more and more present in our private and professional environments in all fields: logistics, industry, agriculture, building, health and even space.

## 1. Introduction

The Internet of Things (IoT) paradigm is about connecting a huge number of devices through various access technologies to exchange information and communicate. The IoT aims to enable multiple functionalities such as identification, location, tracking, monitoring and management in various contexts: privacy, smart homes, industry, health, wearables, 5G/6G mobile communications [1,2,3], and more recently, the use of connected thermal cameras to detect potential COVID-19 infections and sensors to monitor office occupancy levels. Related studies have shown for a few years now that the ubiquity and penetration of IoT will be enormous. The COVID-19 crisis has even generated a wave of digital transformation that will further accelerate its development in the coming months and years, so much so that the IoT market is expected to exceed one trillion by 2024. 

Faced with such a craze regarding the technological challenges to be met, the application aspects that were expected and the potential revenues that would be generated, IoT systems have spread very quickly and continue to develop even though several fundamental aspects were initially “forgotten” and/or are emerging. 

First of all, there is not yet an international standard dedicated to IoT that would allow a better technological harmonization and then further accelerate the deployment of connected objects all over the world. Unfortunately, this observation is repeated again and again. Economic pressures mean that products are put on the market as soon as possible and without any international harmonization. Thus, and this is particularly true in the field of wireless technologies, regulations, when they are put in place, are either an afterthought or the devices are endowed with a certain complexity, useless from a “pure” technological point of view, but which allows portability in different geographical areas of the world. For example, the deployment of ultra large band (UWB) technology [4] is an emblematic illustration. In addition to the industrial conflicts opposing the defenders of multi-band OFDM (orthogonal frequency division multiplexing)-type approaches and those defending the original idea, i.e., exploiting new waveforms of the pulse type, the difficulties in agreeing on the regulation of the radio spectrum at the international level have been a brake on its development; the frequency band authorized by the FCC (Federal Communications Commission) in the United States, a band between 3.1 GHz and 10.6 GHz [5], has been greatly reduced elsewhere in the world, without harmonization, and this has called into question the very interest in the use of very narrow pulses. More recently, the opening of new frequency bands dedicated to UHF (ultra-high frequency) RFID (radio frequency identification) technology in Europe illustrates this phenomenon with regulations that vary from one country to another, knowing that regulations for this technology are already different from one continent to another. With the congestion of the radio frequency spectrum, specific to each country, it has therefore become extremely difficult today to find common international frequency ranges that are available, although this is a real issue. A second weakness concerns security, privacy and data protection. As before, although the problem is known in several other contexts, the “need” (for economic reasons) to bring connected devices to market has not allowed for upstream regulation on these security aspects. Whether IoT systems are public or enterprise, the unstructured and fragmented nature of security regulations is and will be a barrier to their unanimous adoption. Finally, in addition to this lack of standardization, current IoT ecosystems still lack real-time intelligence, which relies heavily on edge computing and the artificial intelligence of objects. However, given the emergence of artificial intelligence in all domains, there is no doubt that this gap will be quickly filled and that this aspect will remain an area for continuous improvement.

By analogy with the well-known OSI (open systems interconnection) model, the IoT can be seen as consisting of five main layers: the sensing layer, the access layer, the network layer, the support layer and the application layer. Thus, the IoT includes multiple technologies: nanotechnologies, sensing and identification technologies, network communication technologies, data fusion technologies, cloud computing technologies, and so-called smart technologies.

In this article, we will focus on sensing, and more specifically on sensors based on RFID technology. The objective is both to highlight UHF RFID and its concepts in a synthetic way, and at the same time to describe how this technology is evolving in recent years in terms of new capabilities (with a focus on sensing) by giving examples of its applications and elements of perspectives. The paper is organized as follows. Section 2 is dedicated to a presentation of RFID, in particular UHF RFID and its principles, and it is also shown why RFID is a promising and relevant technology for the future development of the IoT. Section 3 presents the principles of RFID sensor tags. It is not a question of an exhaustive presentation of various state-of-the-art sensors, because this would be in vain as they are so numerous, but rather to underline on the one hand the main concepts on which they are based and, on the other, to give some examples and emblematic uses. Section 4 draws conclusions and presents perspectives.

## 2. RFID Technology: From Identification to Sensing

Since the advent of the electronic article surveillance (EAS) anti-theft system, which is still widely used, the first emblematic commercial application of which appeared in the 1970’s, RFID technology has found many applications in a wide range of fields such as industry [6,7,8,9,10,11], agriculture [12,13,14], health [15,16,17,18] but also in our everyday life [19,20,21]. At the same time, its applications continue to diversify. From identification (its original function), RFID is used, for example, for access management, logistics and tracking of goods, people and animals, locating lost luggage in airports, and timing of sporting events. In recent years, a major development has been the integration of the sensor function within the tag. It has allowed an extension of the field of applications of RFID, for example, the monitoring of machines or perishable goods, and especially sensor networks and the IoT.

After having recalled the principles and the specificities of RFID technology, in particular the case of passive RFID, it will be shown why RFID proves to be a relevant choice to deploy the first (identification but also detection or recovery of information) or the last meters (identification but also control or command) of the IoT.

### 2.1. A Brief History of RFID

Today RFID technology is already ubiquitous in our daily lives and many people use it on a daily basis without even knowing that it exists. Transport tickets, payment systems, electronic tolls, passports, car keys, and access cards are examples of its application.

However, its development has been lengthy and RFID can be considered as an old technology dating back about 70 years. Indeed, the first applications, based on the physical principles that founded RFID, date back to the 1940s with the identification transponders of British aircraft in the Second World War [22] and the fundamental article of Harry Stockman on the principles of modulated backscattered communication [23]. At the same time, Leon Theremin invented the first passive RFID tag device known as the Great Seal bug, which was designed to spy on what was said at the U.S. Embassy in the Soviet Union [24]. RFID then remained discreet in its application and in industrial fields as well as in the academic field, even if studies were carried out with a few patents to support them. Thus, RFID systems have long been restricted to specific applications mainly in the military field with access control to sensitive sites until the late 1970s, and also the tracking of train cars. These systems were then developed in the private sector with, in particular, the identification of cattle in Europe and use in the production lines of car manufacturers. It is only in 1983 that the term RFID appears in Charles Walton’s patent [25]. In the 1990s, the first efforts at standardization of RFID equipment and, in parallel, the important progress of the integration of the electronic components, led to the development of RFID at the beginning of the 2000s with, in particular, the EPC standard, the international standard of the traceability of objects.

This evolution of interest in RFID in the academic field is very visible when we consider the publications on this technology: almost none until 1995; very few (a few dozen) until 2004, before the MIT’s Auto-ID Center became EPCglobal, an organization whose purpose is to promote the EPC standard, a standard developed by academics and adopted by industry; from hundreds to thousands per year since.

These last few years, the evolution of RFID has notably been motivated by two applicative stakes. It is a question of applying RFID and its identification function, always with traceability as a motivation, but to more and more diverse applications which require specific strategies adapted to particular so-called severe environments (for example, complex propagation channels with multiple paths, stacking of objects to be traced, increasingly miniature objects, presence of humidity, new environments such as space) and with increasingly important performance requirements (all objects must be detected without fault). In parallel to this evolution, linked to the application contexts, RFID is being endowed with new capabilities, with the need to improve security and privacy aspects, and also, notably, the growing development of sensor tags. For example, it is not only a question of ensuring the traceability of a product, but also of making sure that the cold chain has been respected during its transport, which requires detecting the temperature or at least detecting whether a temperature threshold has not been exceeded. This new capability associated with RFID extends its field of applications in an extremely significant way and motivates a large part of the current work carried out in this field. It is remarkable to note that as early as 2004, Roy Want published an article [26] along these lines, writing that “in the near future, RFID tags will be widely used as environmental sensors” while already citing some examples. It is also possible to wonder about the fact that RFID could one day replace the traditional barcode as was often heard at the time of its rise; the idea of being able to pass directly through checkout with a complete cart with the purchased products.

In terms of impact on the economic and social domains, despite the economic recession of 2008, and the COVID-19 pandemic, the RFID market continues to experience double-digit annual growth, amounting to $7.67 billion in 2012 and expected to reach $70.5 billion by 2022 [27,28]. For example, RFID technology was already present in the 34 priority plans of the new face of industry in France presented in September 2013.

Before seeing how tags have evolved into sensor tags and which sensor tags are encountered today, the following section summarizes the principles and characteristics of RFID technology and highlights its advantages.

### 2.2. A Glimpse of the Variety of RFID Technology

RFID is an automatic wireless data-collection technology very popular in different applications and services including logistics, manufacturing, access control and security. RFID technology has always been part of the « top ten » technologies worldwide. This growing interest is primarily related to the remarkable benefits of RFID, standardized communications enabling inter-operability (according to the local regulations either from ETSI in Europe or the FCC in US, with well-defined protocols, mainly ISO and EPCglobal), and, in particular, its passive and wireless features that provide decisive practical advantages: no maintenance and a practically unlimited life. As evoked above, the scope of RFID technology is nowadays not only limited to the identification and tracking of inventory; its potential to collect and compile massive amounts of detailed real-time data about the environment around us (including human body characteristics) opens the way for a plethora of new applications in the area of smart skins [29], man-to-machine, cognitive intelligence [30], and especially the IoT.

The general principle of an RFID system is based on one (or even several) readers capable of reading tags (or so-called “smart” labels), which are attached (depending on the case, glued, sewn, inserted, etc.) to an object, an animal or a person, and present in the reader’s environment. The reader itself is usually connected to a base station acting as a database. The reading consists in retrieving the tag’s identifier, or even in writing information in the tag which can also be read.

**Figure 1:**
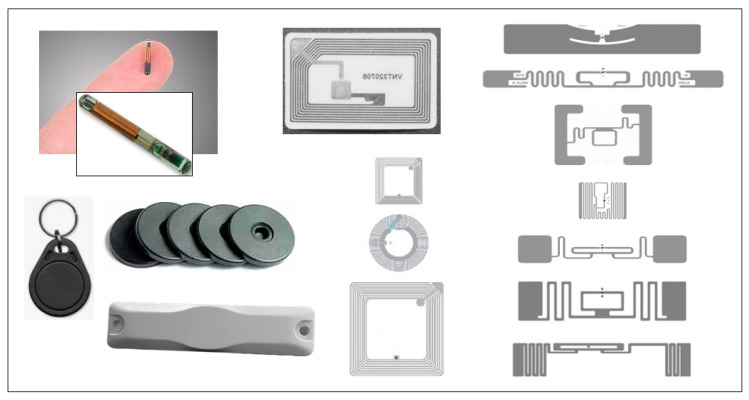
Examples of different types of tags.

There are many types of tags, as shown in Figure 1, whose shapes and dimensions vary according to the standards (directly related to the frequency used).

**Figure 2:**
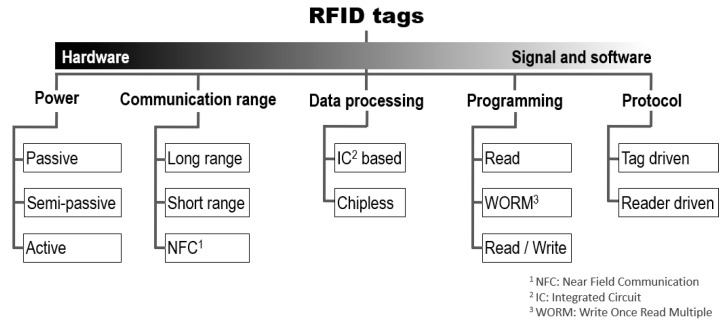
Classification according to several criteria of the different families of tags.

To classify the many different types of tags, which underlie the different types and even standards of RFID technology, it is possible to adopt the general taxonomy presented in Figure 2. Power, communication range, data processing, programming, and protocol are different parameters that allow us to classify the types of RFID systems, considering aspects ranging from hardware to signal and software.

It should be noted, however, that a classification of tags is more complex and difficult to explain and it is therefore necessary to cross-reference information to be more precise. Indeed, a passive tag, for example, may be specific to applications with greater or lesser distances involved, a passive tag may or may not include a chip, or it may incorporate several different programming mechanisms. However, categorizing the tags allows us to highlight their main characteristics.

In addition to Figure 2, it is interesting to cross-reference the given information with the various existing standards which depend essentially on the frequency ranges used, and of which it is possible to find detailed complements, for example, in [31,32,33].

- LF (low frequency): frequencies between 125 and 134.2 kHz. The maximum detection range of a tag responding to this frequency is about 50 cm. The characteristics associated with this frequency range are: high price even with large volumes, low impact of a metallic or liquid environment on reading performance. There are several standards (ISO 18000-2, ISO 11784, ISO 14223, etc.) but the frequency ranges used are the same all over the world. This standard is used, for example, for animal tattooing or car keys.
- HF (high frequency): frequency of 13.56 MHz. The maximum detection range of a tag responding to this frequency is about 1 m. The characteristics associated with this frequency are as follows: lower price than LF tags, suitable for applications requiring contact reading without a large volume of tags to be read, global frequency (the same in all countries). Examples of applications are access control or electronic passports. Note that NFC (near field communication) belongs to this category.
- UHF (ultra-high frequency): frequencies between 864 and 928 MHz [34]. The maximum detection range of a passive tag in this frequency range is approximately 3 to 20 m, depending on the propagation conditions. The characteristics associated with this frequency range are as follows: lower price than LF and HF tags for large volumes, suitable for applications requiring reading distance and a large volume of tags to be read very quickly, tags dedicated to constrained environments (metal, liquid, etc.). The uses of this standard are, for example, logistics, item identification, traceability, tolls.
- SHF (super-high frequency): frequencies between 2.45 and 5.8 GHz. The maximum detection range of an active tag in this frequency range is about 100 m. The characteristics associated with this frequency range are as follows: relatively similar performance to UHF, high sensitivity to metal and liquid environments, directionality of tag detection. The frequency range in which SHF RFID systems operate are those which are globally unlicensed, allowing these systems to be used globally. However, these frequency bands are crowded and can be prone to interference as many devices such as cordless phones and microwave ovens use these frequencies.

It is also important to highlight the distinction between passive and active tags. A passive RFID tag, as its name indicates, is purely passive, i.e., it does not integrate either a battery or a radio frequency transmitter. A passive tag uses the wave (magnetic or electromagnetic) from the interrogator (RFID reader + antenna) to power the embedded electronic circuit (i.e., its integrated circuit, IC, called the chip) and allows it to communicate the information contained in its memory by using the backscattering principle. A semi-passive RFID tag (or battery-assisted passive, BAP) has an integrated power supply (batteries). The power supply is not used to provide energy to a radio frequency (RF) transmitter since the communication principle is the same as that of the passive RFID tag. This energy is used to power either the electronic circuit of the tag (the activation of the tag no longer relies on remote powering via the reader, which can increase the reading distances) or a sensor (e.g., temperature, current, acceleration, gyroscope, etc.) connected to the RFID chip. These tags require maintenance related to the change of batteries. An active RFID tag integrates an RF transmitter and thus a power supply (batteries). Like semi-active tags, it can be equipped with sensors and can, for example, embed an additional microcontroller to ensure its own signal processing. This tag can interact autonomously with its environment thanks to its battery: sending its position, taking temperature, etc. They also require maintenance related to the replacement of the battery, more frequently than for a semi-active tag.

Today there is also another type of tag that is completely passive but does not include a chip, which is called a chipless tag [35]. The working principle mainly relies on the complex signals emitted by the reader and the way the reflective structure of the tag reflects the transmitted signal (frequency coding, phase coding, etc.). Aside from the absence of a specific regulation standard, the frequency range often used is that of the ultra-wide band technology. The potential of attractive chipless approaches is limited, however, by the complexity of the propagation channels that significantly reduce its read distance and coding capability. Furthermore, since chipless tags are linear scatterers, like any other object in the environment, these limitations cannot be compensated for by increasing the power or sensitivity of the reader since the increase affects the tag and objects in the same proportion [36]. However, a great deal of work is currently being carried out on this technology, which has the immense advantage of using tags that are not only passive but also devoid of any electronics. There is no doubt that this technology will find its own applications in the near future.

### 2.3. Focus on UHF RFID Based on Backscattering

One of the major barriers to the deployment of IoT technologies was, and perhaps still is, the need for an independent power source for sensor nodes; a constraint that increases complexity and cost but also hinders the convenience of deployment and maintenance. However, with recent advances in ultra-low power electronics, new lithium-ion battery technologies, and the implementation of ultra-low power wireless communication standards, the emergence of the IoT is now a reality. Still, the traditional sensor node architecture requires an independent power source and therefore cannot be considered for long-term, maintenance-free sensor networks.

For these reasons, the rest of this presentation focuses mainly on passive but also semi-passive UHF RFID (the latter presenting a relevant compromise in the case of sensor tags as will be shown in Section 3). Indeed, active tags are more similar to traditional wireless technologies where each node integrates a radio transmitter and receiver. Moreover, chipless technology remains very specific, and will not be considered here either. It should be noted, however, that today it also integrates information capture capabilities in addition to the identification function. A state-of-the-art example can be found in [37]. It should also be noted that some work has shown that it can be used in the traditional UHF RFID bands [38]. To more positively justify the focus of this presentation, it must be said that detection techniques based on passive UHF RFID technology have gained in attractiveness in recent years. Indeed, passive UHF RFID tags have many advantages: they are cheap (a few cents to tens of cents in euros), easy to use (light, sometimes self-adhesive) and, especially, relatively non-intrusive with the double advantage of being wireless and battery-free. However, as will be seen later, most sensors based on RFID tags require significant customization of the tag, which increases their cost and size.

**Figure 3:**
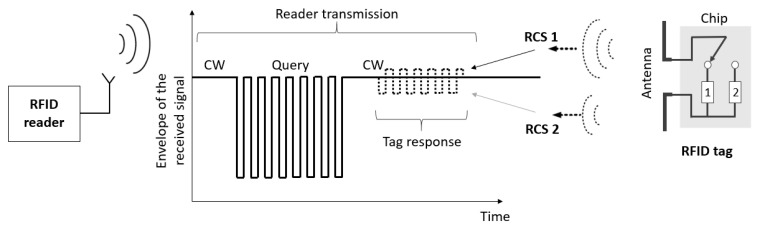
Principle of a passive RFID system.

In passive UHF RFID systems, as already highlighted, the tag does not have an autonomous energy source like a battery, but is power supplied by the reader via electromagnetic fields. The principle of the passive UHF RFID system is illustrated in Figure 3. The reader generates a carrier wave (CW) that is transmitted by the reader antenna. Reader antennas are typically directive, i.e., they illuminate only a certain volume, the so-called read zone. If a RFID tag (that is constituted by an antenna and a chip) is inside the “read” zone (or interrogation zone), the power transmitted by the reader activates the tag and it is ready to receive commands (so-called Query). The EPCglobal Class-1 Gen-2 is a reader-talks-first protocol, i.e., the tags wait until they are addressed. Upon receipt of a command, which is transmitted via modulation of the CW, the tag sends its identification code or parts of its memory content. The tag does not actively transmit data, but it reflects part of the incident reader carrier wave by deliberately de-tuning its own antenna generating two different radar cross-sections (RCS) as depicted in Figure 3. This principle is known as backscatter modulation [39,40].

Compared to other RFID standards, the passive UHF RFID systems offer relevant solutions because of long autonomy, read range, size of tags and the capability to read several tags in the same time because the reading protocol is based on an anti-collision algorithm. UHF RFID offers connectivity to a widely used frequency band (860–960 MHz) according to the local regulations with well-defined protocols (mainly ISO, EPCglobal), achieving reading range in some cases above 10 m.

The readability strongly depends on the propagation environment and on the object (i.e., its composition and matter) attached to the tag. These variations depend on dielectric constant parameters such as the relative permittivity, the relative permeability, the conductivity, the magnetic loss factor, the mass density, but also physical characters such as dimensional constraints and volume. However, these properties also allow the possibility for using modifications in the response of an RFID tag as a sensing mechanism by correlating a change in some physical parameter of interest.

**Figure 4:**
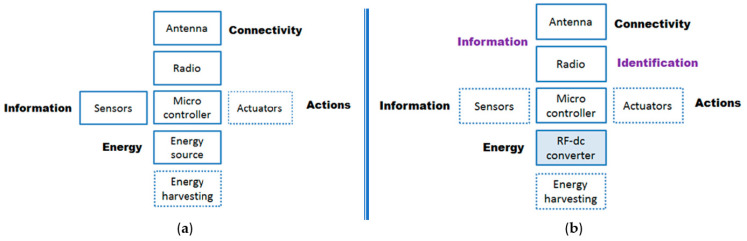
Comparison between architectures: (**a**) Low power sensor technology. (**b**) Passive UHF RFID sensor technology.

Figure 4 illustrates the functional architectures of low power sensor technology and passive UHF RFID sensor tag technology highlighting the differences and possible options in the RFID case (note that the functional blocks circled in dotted lines in the figure suggest the optional side of their presence). As illustrated, the global architectures are generally quite similar. A microcontroller manages and drives the other functional blocks (including the memory, not shown here):

- Connectivity (i.e., exchange of information by wireless link): a traditional sensor integrates an RF transmitter–receiver module, whereas a tag relies on communication by retro-modulation;
- Energy (i.e., power source): a traditional sensor integrates a battery while a tag has a dedicated RF–DC converter; and, in both cases, an energy recovery unit can be added.
- Information (i.e., data): for a traditional sensor, the information transmitted is that captured or detected by the dedicated part; in the case of a sensor tag, the data includes the identifier (ID) but also the information collected, which may come either from a sensor associated with the tag (similar to the other case) or intrinsically from the tag as evoked above, e.g., a tag whose substrate (but also the antenna or the chip) is voluntarily made sensitive to the magnitude to be captured and then implicitly allows the information to be transmitted during retro-modulation.
- Actions: in one case as in the other, an actuator can drive a particular function, for example, to activate an in-switch, a luminous indicator (LED type), a sound signal, etc.

The main differences are therefore in the power supply, the data transmission and the intrinsic identification functionality. In addition to the advantages and possibilities of the RFID approach, which will be detailed later, it should be noted that the classical sensor architecture is more versatile because it offers a greater degree of freedom and allows the interconnection of various modules quite easily; thus, it is possible, for example, to choose the radio technology, to add an additional energy-independent block (sensor, actuator, memory, etc.), or to perform (pre-)processing within the sensor itself. The addition of the sensor function in a passive RFID tag is an option that requires a specific realization. For an active RFID tag, the architecture is more similar to that of a traditional wireless sensor (including a battery and a modulator) and in this case the identification functionality is obviously present.

### 2.4. Other Possible Arguments in Favor of RFID

Without getting into long polemics, RFID also has its detractors denouncing, on the one hand, the non-respect of privacy by its inclusive character and, on the other hand, the risk of exposure to electromagnetic waves. Without wanting to take sides and open a debate on this subject which would exceed the contents of this article, it is possible here to give some arguments in favor of RFID in a context of emergence of the IoT.

First of all, some technological elements can be put forward which demonstrate that RFID can contribute, in comparison to other wired or wireless technologies, to safeguard a “green” world in a context where few people are ready to do without advanced technologies and more specifically connected objects. The wireless character translates into electromagnetic waves but is freed from using cables. The passive character means that there is no battery in the tags, which means that no maintenance is required and no batteries are used. Furthermore, this constraint implies very low consumption devices (about 10 µW are required to power a tag), and also, the communication protocol is generally based on a LBT (listen-before-talk) protocol which implies that the tags are 100% switched off when they are not communicating. It is also interesting to note the significant progress made in recent years in terms of tag sensitivity (or activation power): in 1997, the best sensitivities were around −8 dBm, whereas today they reach nearly −27 dBm. As a consequence, the theoretical reading distances (i.e., in free space and using the maximum authorized power) have increased from 5 m to nearly 40 m. Thus, for the same application, the power transmitted by the reader to ensure the interrogation can be decreased. In addition, to maintain this passive character, new solutions consist in equipping the tag with an energy harvester, often electromagnetic (but there are other exploitable energy sources such as mechanical, thermal, solar, etc.) in order to either pre-activate the tag (powering the integrated circuit) or to power the sensor(s) associated with the tag. Moreover, the trend is also to use natural materials as a substrate (support on which the antenna and the chip are placed to constitute the tag).

On the other hand, some of the applications of RFID aim directly or indirectly to reduce the carbon footprint in a number of areas of human activity; for instance, improving recycling through refuse management, reducing vehicle emissions through better usage, improving the management of natural resources, encouraging the re-use of containers, tracking animals to monitor the impact of climate change, and reducing equipment by better asset management [41].

The traceability functionality of RFID has also generated and motivated the numerous attacks against this technology. This is a point that can be seen positively because as a consequence RFID is probably the most advanced technology on this kind of questioning as demonstrated, for example, by the reflections and reports on RFID and privacy established by the European Commission, as well as by industrial actors and academics [42].

Another important note is that passive UHF RFID technology is now better known in the industrial world as RAIN, derived from “RAdio frequency IdentificatioN”. It is an alliance of industrial players that aims to promote the widespread use of passive UHF RFID, specifically the UHF Gen 2 standard (ISO/IEC 18000-63). Under this term, there is the idea of a cloud-based infrastructure, where data is stored, managed, and shared over the Internet, data coming from RFID products; which is perfectly consistent with the IoT. Moreover, there is also a certification called RAIN RFID, which allows the proposal of “universal” solutions (RAIN certified), which integrate all tags, readers, connectivity and software. RFID technology, especially passive RFID, appears to be a technology with great potential for the future, and the IoT in particular. It will be imperative to ensure that its development is achieved while respecting the essential pillars of our social pact in terms of freedom, health and environmental protection.

## 3. RFID Sensor Tags

### 3.1. Quick Look at the Types of RFID Sensor Tags That Exist

Today, there is a plethora of RFID sensor tags, at least in the literature, because commercial solutions, if they exist, are still few. Generally speaking, like any sensor, an RFID sensor tag is able to detect changes and events, and they are useful in all situations where measurements need to be captured remotely and automatically.

Tags could detect changes in movement, stress, strain, vibration, tilt, etc., often to control the state of a structure, as varied as that may be (tightening of a screw, building, aircraft wing) [43,44,45,46,47], humidity, temperature, moisture [48,49,50,51,52,53,54,55], corrosion and chemicals [56,57,58,59,60], and also pressure, light level, audible feedback, or biometric data, etc. Note that most of these measures can be absolute or relative, for example, to detect the passage of a threshold.

It is also interesting to note the use of specific materials when the tag becomes a sensor tag (without added sensors). For example, for temperature or humidity the information capture can be based on relatively simple to use materials such as polyvinyl-alcohol (i.e., PVA) polyimide film (i.e., Kapton). Even if the context of the article [61] aims at chipless solutions, the principles being ultimately quite similar, many examples of materials that can be used, associated with their different characteristics, are given.

In addition to these rather classical sensor applications, the potential field of use is extremely vast and will only grow in the future. For example, we can cite recent and original studies that have focused on ice detection [62], solutions for metallic environments integrating both European and American standards [63], and even the use of RFID to monitor the health of astronauts [64].

Some authors refer to this type of tag as an “augmented tag”, whose capabilities then go beyond the mere identification functionality, in this case, with the sensor function being the most frequent function [65,66].

The second part of this section presents the main approaches to transform a tag into a sensor tag, and also gives some elements of comparison with other techniques dedicated to passive wireless sensing. With an applicative look, specifically considering health, the third part aims to illustrate and show in a given context the types of sensors implemented.

### 3.2. Principles of RFID Sensor Tags

There are two main types of architectures to design an RFID sensor tag [67]: either the tag integrates one (or more) additional sensor, an independent sensor that is connected to the tag or even integrated into its chip, or the sensor function is integrated into the tag by a judicious design adding to the chip but more often to the antenna the role of sensor by also using it as a transducer [68,69,70,71].

In the case where the tag is associated with a sensor, one of the main limitations lies in the ability to maintain the passive nature of the tag and sensor assembly. It is necessary to power the attached sensor autonomously: either by using the rectifier circuit of the tag itself, but this is then to the detriment of the tag’s performance (part of the energy is diverted), or by using a dedicated energy recovery device that exploits the energy from other sources such as solar, thermal, kinetic or electromagnetic [71,72,73,74]. The other solution, which is technologically simpler, is to add an onboard battery dedicated to the sensor part at the expense of the 100% passive character. Based on this approach, several sensor-tag platforms are now available such as the well-known wireless identification and sensing platform (WISP) which is a programmable, microcontroller-based sensor tag, compatible with the EPCglobal UHF RFID standard [75] and comparable platforms from academic labs [60,76] and commercial manufacturers [77,78,79]. This principle allows a priori better performance in terms of measurement ranges and accuracy, or at least allows the capabilities of the sensor associated with the tag to be fully exploited.

The second approach, which consists of inserting the sensor function into the tag itself, is an ingenious alternative that makes it possible to preserve the passive character. However, it suffers from the fact that the characteristics of the backscattered signal are altered in terms of the detection of information (identifier + sensed quantity), which reduces the reading range and even the reading capacity. To overcome these problems, solutions aim at dividing and/or modifying the coding of the information returned by the tag in order to separate the two information channels (identification and detection), for example, phase modulation for the sensor and amplitude modulation for the communication or specific modulation frequency for the sensor [80]. A hybrid analog–digital platform has also been proposed that uses digital backscatter for addressing and control, and an analog backscatter mode for high-speed transmission of sensor data [81].

More generally, techniques exploiting RFID to generate sensors are also constrained or limited by time factors, for example, the time needed to acquire data while the device is in motion [82]; or the variation of the phase of the signals (a property exploited for localization purposes), which it is a priori necessary to overcome for the purposes of capturing information (eliminating any calibration) [83].

It is also worth noting that due to mass production printing techniques and the advances in fabrication of integrated circuits, the cost of a RFID tag can be very low, less than $0.10. However, RFID tags with more capabilities and complexity can cost more than $100. This is why it seems more judicious to design simple RFID sensor tags which are not, for instance, microcontroller-specific in order to manage sensing operations or specific connections for external sensors. Generally, in the field of RFID, it is more interesting to keep the tags simple (with an attractive cost) and to put the complexity on the side of the readers (which can be a little more expensive and which, moreover, are not—or are less, in the portable case—confronted with the problem of power supply).

To summarize and position the approaches used for RFID sensor tags more globally compared to other techniques, Table 1 (which comes from the reference [80]) compares five different techniques for providing a wireless sensor; the two right-hand columns correspond to the techniques presented here, which are based on RFID with a tag that integrates a digital sensor or exploits the sensitivity of the antenna. The comparison criteria put forward are very relevant and distinguish the intrinsic characteristics of each technology.

If there is a lot of research work on RFID sensors, the commercial availability of these RFID sensor tags is still quite limited, which shows that technological advances are still expected to facilitate their manufacture in large numbers, to promote their deployment and limit costs.

### 3.3. Focus on the Use of RFID Sensor Tags for Health

To illustrate the evolution and variety of RFID sensor tags, this part focuses on RFID applications in the health field. Indeed, the healthcare industry [84] and academic research [15,16,17,18,85] reflect very well the ever-increasing interest of this technology and its contributions. In other words, the healthcare field is representative of the diversity of passive RFID sensors and the variety of their applications, so it is highlighted here. Moreover, a proof of this fact is that tagging medical instruments and devices is one of the fastest-growing application areas for RAIN RFID in healthcare.

The healthcare sector already relies on a number of RFID applications:

- The identification of people, which means that throughout their journey, and whatever the length of their stay, patients are identified, and even located, especially at-risk patients who do not have authorization to leave; moreover, all care, prescribed treatments, and consumed drugs are automatically recorded.
- The identification of medical files, allowing for their traceability in order to ensure the management, archiving and storage aspects automatically and efficiently, for greater security.
- The traceability of organic tissues, samples and blood products is also automated, and consequently, made reliable.
- The management of large equipment and their maintenance in operational condition is also simplified; they can be located with a follow-up of their state (for example, ready for use, not cleaned, in service or not); the traceability of equipment throughout their life cycle is also favorable to the planning of renewals, investments, and even recycling procedures.
- Inventories, stock management and procurement are also greatly simplified, whether for drugs or medical prostheses, but also for small equipment (syringe pumps, syringes, surgical tools, etc.).

In the medical field, there are two main families of sensors depending on the positioning of the sensor which is located outside the human body (on an object, an equipment, on the person; in this last case, we find the notion of wearables, which is strongly developing today, in the medical field but also for monitoring people in dangerous environments, for example, and even for the leisure of individuals) or inside the human body. In this last case, from a technical point of view, compared to other types of applications, it is necessary to design adapted implantable antennas [86,87] and consider the specific propagation channel [88]. In the first case, a device outside the human body, the main technical specificity is where the device is still on a human body and we then find ourselves with the same issues as those of the wearable. It is then the antennas on textile support or adapted to textiles need to be developed [89,90], and in the case of RFID there is a need to integrate the sensor function [91,92,93].

Finally, more than ever with these types of wireless communication applications, it is advisable to adopt a precautionary principle, especially for fragile people (such as newborns) with respect to exposure to electromagnetic fields, the effects of which are still poorly known, even if regulations exist [94].

## 4. Conclusions and Perspective

RFID is emerging as one of the key technologies for e-commerce, paperless business, ubiquity of sensors, autonomous sensor networks, ambient distributed intelligence and the IoT. Indeed, RFID relies on well-established wireless communication standards (ISO 18000-x) that can be easily connected to wireless or wired network infrastructures for a larger spatial scale. In particular, passive UHF RFID relies on back-modulated (or backscattered) communications, and as a result, the associated tags are passive (or even semi-passive) and therefore do not embed a power source. This fundamental characteristic ensures autonomy, indirectly limits their weight, and it also allows the devices to be free of any maintenance, and implicitly allows a potential recycling of the batteries. Moreover, tags nowadays integrate a sensor function and are able to detect multiple types of physical quantities. While academic research is increasingly rich in this field, commercial tags are still quite rare but some are already deployed. For example, the SL900A EPC Gen 2 sensor tag offers the following applications: supply and cold chain, tracking condition and history of constructions, tire pressure monitoring systems (TPMS), and contactless metering.

Note also that besides the new challenges necessary to support these new functionalities (such as sensing or localization), passive UHF RFID still has its two historical limitations: (i) the read range, i.e., the maximal reading distance between reader and tag allowing both the tag activation (wireless power transfer) and the tag-to-reader information transfer via backscattering, and (ii) the inventory rate, i.e., the capacity of the reader to identify all the tags in its vicinity. Although significant progress has been made in recent years in terms of chip sensitivity, chip impedance self-tuning, specific tag and/or tag antenna conditioning, near-field reader antenna, etc., performance remains highly dependent on the application (type of objects tagged) and the environment (complexity of the propagation medium and multipath).

RFID tags are expected in the coming years to act as input–output access points of a heterogeneous data capture infrastructure, which will enable very efficient applications through data aggregation, all with a remarkable power consumption control (tags are passive devices) and a reduced hardware footprint (tags are wireless devices).

Additional functions or capabilities are expected in the short term, such as: miniaturization of tags for increasingly small objects, actuating operations, applications in harsh environments, security and authentication, and localization (which remains a hot topic in the RFID field). In the current application context, it should be noted that all OSI layers are concerned, physical, middleware and application layers.

Concerning security aspects of RFID tags, solutions based on the coupling of lightweight cryptography and the physical fingerprint (such as physical unclonable function and/or electromagnetic signature) will certainly see the light of day to overcome the limitations of tags in terms of computing resources, area resources, and power budget [95,96]. In the longer term, it is likely that new standards will appear such as chipless (already mentioned), but also MMID (millimeter-wave identification) of which several prototypes have been proposed including the sensor function [97]. The use of specific waveforms (notably pulsed-mode) in the RFID domain is also a widely explored track because it allows optimization of both the energy transfer (for example, for remote powering) and the information transfer [98,99,100]. However, the deployment of such approaches requires more versatile readers, capable of generating arbitrary signals and therefore more expensive at the moment. However, in addition to the advantages in terms of performance, these approaches could also adopt current standards and thus not require specific standardization. Note also that multi-standard sensor tags are likely to be developed in the future [101]. The concept of tag-to-tag [102,103] should also eventually break through, offering the possibility of tags communicating with each other and thus imagining new architectures of sensor networks with tags communicating, exchanging information, sharing resources, etc.

## Figures and Tables

**Table 1:** Comparison of the passive wireless sensors features [80].

	Resonance Sensor	SAW ^1^ RFID	Intermodulation Sensor	RFID + Digital Sensor	RFID + Sensitive Antenna
Communication	Analog	Analog	Analog	Digital	Digital
Sensing	Analog	Analog	Analog	Digital	Analog
ID	No	Yes	Yes	Yes	Yes
Memory	No	No	No	Yes	Yes
Auto-collision	No	No	No	Yes	Yes
Environment intensive	No	Yes	Yes	Yes	No
Read-out distance	Small	Large	Large	Small	Large
Sensing element	Generic	Special	Generic	Generic	Special
Reader device	Special	Special	Special	Standard	Standard

^1^ SAW: Surface Acoustic Wave.
